# The effect of calorie restriction on the presence of apoptotic ovarian cells in normal wild type mice and low-plasma-IGF-1 Laron dwarf mice

**DOI:** 10.1186/1757-2215-6-67

**Published:** 2013-09-24

**Authors:** Sylwia Słuczanowska-Głąbowska, Maria Laszczyńska, Katarzyna Piotrowska, Wojciech Głąbowski, Bogdan Rumianowski, Michal Masternak, Oge Arum, Magda Kucia, John J Kopchick, Andrzej Bartke, Mariusz Z Ratajczak

**Affiliations:** 1 Department of Physiology, Pomeranian Medical University, PowstańcówWielkopolskich 72, 70-111, Szczecin, Poland; 2 Department of Histology and Developmental Biology, Pomeranian Medical University, Żołnierska 48, 71-210, Szczecin, Poland; 3 Department of Histology and Embryology, Pomeranian Medical University, PowstańcówWielkopolskich 72, 70-111, Szczecin, Poland; 4 Stem Cell Biology Program, James Graham Brown Cancer Center, University of Louisville, Louisville, Ky, USA; 5 Edison Biotechnology Institute and Department of Biomedical Sciences, College of Osteopathic Medicine, Ohio University, Athens, OH, USA; 6 Geriatrics Research, Departments of Internal Medicine and Physiology, Southern Illinois University School of Medicine, Springfield, IL, USA

**Keywords:** Ovary, Laron dwarf mice, Caloric restriction, Apoptosis, Aging

## Abstract

**Background:**

It is known that caloric restriction extends lifespan and can minimize age-related dysfunction of the reproductive system. We became interested in how caloric restriction influences apoptosis, which is a crucial process that maintains ovarian cell homeostasis.

**Methods:**

We examined ovarian cells in: 2.5-year-old wild type mice on caloric restriction (CR) or fed *ad libitum* (AL) and Laron dwarf mice (GHR-KO) at the same ages on CR or fed AL. Apoptosis was assessed by histochemical analysis on paraffin sections of ovarian tissue.

**Results:**

Morphological and histochemical analysis revealed that CR improved reproductive potential in 2.5-year-old WT littermates and GHR-KO female mice, as indicated by the increased number of ovarian follicles. The level of apoptosis in ovarian tissue was higher in WT mice on a CR diet compared with WT mice on the AL diet. In GHR-KO mice, the level of apoptosis in ovaries was similar for mice on CR and on AL diets and bigger than in WT mice on CR.

**Conclusions:**

Morphological and histochemical analysis revealed a younger biological age of the ovaries in 2-year-old WT littermates and GHR-KO female mice on CR compared with animals fed AL.

## Introduction

The ovary is a very dynamic structure, and proliferation and apoptosis in this organ are in constant equilibrium, affected by many factors, including hormones and nutrition [1-5].

Dietary caloric restriction (CR) is known to extend lifespan and minimize age-related dysfunction of many organs, including those of the reproductive system [6]. In 1917, Osborne et al. observed that decreasing food intake increased lifespan in rats [7]. Masoro et al. [8] found that a CR diet decreased plasma levels of glucose and insulin, although these animals utilized glucose at the same rate per metabolic mass unit as animals fed *ad libitum* (AL). The authors suggested that CR increases insulin responsiveness and that low glucose and insulin concentrations in plasma extend lifespan.

A direct benefit of a CR diet on reproductive life span has also been demonstrated [4,5,9]. Reproductive senescence in females is measured by the number of primordial ovarian follicles, which may be recruited for further development and maturation [5], and a lower number of primordial follicles is a feature of older ovaries [10]. Selesniemi et al. [11] observed that CR has an influence on the number of oocytes. Morphometric analysis of the number of non-atretic follicles in 8-month-old mice indicated that mice on CR possess almost double the number of primordial follicles and an increased number of primary follicles compared with mice fed AL. At the same time, the number of atretic follicles was reduced in these animals. The effectiveness of CR was also evaluated by weekly monitoring of body weight, which showed that body weight of 8-month-old mice on CR was approximately 25% lower than 8-month-old mice fed AL [10].

Growth hormone receptor (GHR) knock-out mice (GHR-KO), also called Laron dwarf mice, correspond to one form of human dwarfism known as Laron syndrome. These patients have high levels of GH due to a defect in the GHR and a very low level of insulin-like growth factor-1 (IGF-1) circulating in plasma. These patients are dwarfs with marked obesity, delayed puberty [12], and resistance to cancer. Laron mice (GHR-KO) mimic human Laron dwarfism and are characterized as long-living dwarf animals with high levels of GH and very low levels of plasma IGF-1. Bonkowski et al. in the longevity study of Laron dwarf mice observed lack of response to CR in context of increase of lifespan in these animals. Authors suggest that longevity depends on insulin sensitivity which cannot be further increased in GHRKO mice [13,14]. These mice are fertile but display delayed sex maturation [15].

Our recent investigation revealed a less advanced biological age of ovaries isolated from 2.5-year-old GHR-KO mice compared with ovaries of the 2.5-year-old wild type (WT) littermates. The ovaries of GHR-KO mice were smaller but had normal structure, including typical cuboidal epithelium on the surface, and contained primary, preantral, antral, and Graafian follicles [10].

We became interested in how CR influences ovarian apoptosis in these animals. Apoptosis is detectable in human and animal ovaries throughout fetal and adult life [1,16,17]. It is believed to be a crucial process for maintaining ovarian homeostasis, and during life, most of follicles undergo apoptosis [18]. In fetal life, apoptosis is mainly confined to oocytes; however, in adult life it is found in granulosa cells of preantral and antral follicles and in theca cells, both in humans and in other mammals. Apoptosis limits the number and development of ovarian follicles in the menstrual cycle, so that only one or a few follicle-enclosed oocytes will reach the stage of Graafian follicle and ovulate, and this process is essential for preventing multiple embryos during pregnancy. Apoptosis causes follicle atresia before they become capable of ovulation and is responsible for regression of the corpora lutea. The apoptotic process in corpora lutea is crucial for preserving cyclicity and for ensuring the release of progesterone during the menstrual cycle [1,16,17,19-21]. Hułas-Stasiak et al. [17] observed that both autophagy and apoptosis are involved in follicular atresia in mice. While autophagy is responsible for follicular atresia at the moment of birth, apoptosis is the dominant form of postnatal ovarian atresia [17]. Interestingly, Danilovich et al. [22] postulated that GH and IGF-1 prevent the apoptosis of granulosa cells.

The aim of this study was to examine the influences of CR on ovarian apoptosis in GHR-KO mice, with low circulating plasma levels of IGF-1, compared with age-matched WT animals.

## Materials and methods

### Animals

Two and half year-old female mice were used in our experiment, including GHR-KO Laron dwarf and WT animals.

We employed female Laron dwarf (growth hormone receptor, GHR^−/−^, also known as GHR-KO) mice derived from a population produced by crossing 129Ola/BALB/c GHR^+/−^ animals (generously provided by Dr. J. J. Kopchick) with mice derived from crosses of C57BL/6 J and C3H/J strains and normal (wild type) animals from the same strain. These mice were produced at the animal facility at Southern Illinois University of Medicine School [15] from a closed colony with inbreeding minimized by avoiding brother × sister mating. The experiment were conducted with approval of Southern Illinois University School of Medicine Protocols. Protocol number 178-02-001.

### Experimental design

The female mice were divided into four groups. We compared WT mice (n = 10) on a CR diet (WT-CR) to WT mice (n = 10) on a normal AL diet (WT-AL) and Laron dwarf (GHR-KO) mice (n = 10) on a CR diet (GHR-KO-CR) to GHR-KO mice (n = 10) on a normal AL diet (GHR-KO-AL).

The animals were housed under temperature- and light-controlled conditions (20–23°C, 12-hr light/12-hr dark cycle) until the age of 30 months, when the animals were sacrificed and the ovaries collected. The animals on a normal AL diet were given free access to nutritionally balanced diet (commercial chaw) and tap water. The animals on CR were fed with reduced amounts of food (30% CR) every day. The CR was initiated at the age of 2 months.

### Morphological analysis of ovarian tissue

The murine ovarian tissue was fixed in 4% buffered paraformaldehyde and subsequently embedded in paraffin. The ovaries were sectioned at a thickness of 3 μm with a Microtome HM 325. The sections of each ovary were mounted on glass slides, counterstained with hematoxylin and eosin and examined by light microscopy (BX41 Olympus).

### Hematotoxylin and eosin (H&E) staining

For H&E staining, the sections were deparaffinized and rehydrated. The hematoxylin was applied for 3 min, and subsequently the sections were rinsed in tap water for 10 min. Next, the eosin was applied for 30 sec and the slides washed in distilled water, dehydrated, and sealed in mounting medium with a coverslip.

### Histochemistry

#### Detection of apoptosis by the TUNEL reaction (terminal deoxynucleotidyltransferase-mediated dUTP nick end-labelling)

The paraffin sections were mounted on poly-lysine-coated glass slides. After deparaffinization and rehydration, the tissue sections were digested with proteinase K (DAKO), and the endogenous peroxidase activity inhibited with Peroxidase Blocking Reagent (DAKO). Next, in a humidified chamber at room temperature, the sections were incubated with terminal deoxynucleotidyltransferase (TdT) and digoxigenin-conjugated deoxynucleotides for 60 min, and the anti-digoxygenin antibody conjugated with peroxidase was applied for 30 min (ApopTag®Peroxidase *In Situ* Apoptosis Detection Kit, Millipore). Next, diaminobenzidine (DAB) was employed to visualize the reaction. The sections were counterstained with hematoxylin, dehydrated, mounted, and coverslipped. After each step of this procedure, the sections were rinsed with PBS and examined by light microscopy (BX41 Olympus).

## Results

### Ovaries of 2.5-year-old WT mice on AL and CR diets

**Figure 1:**
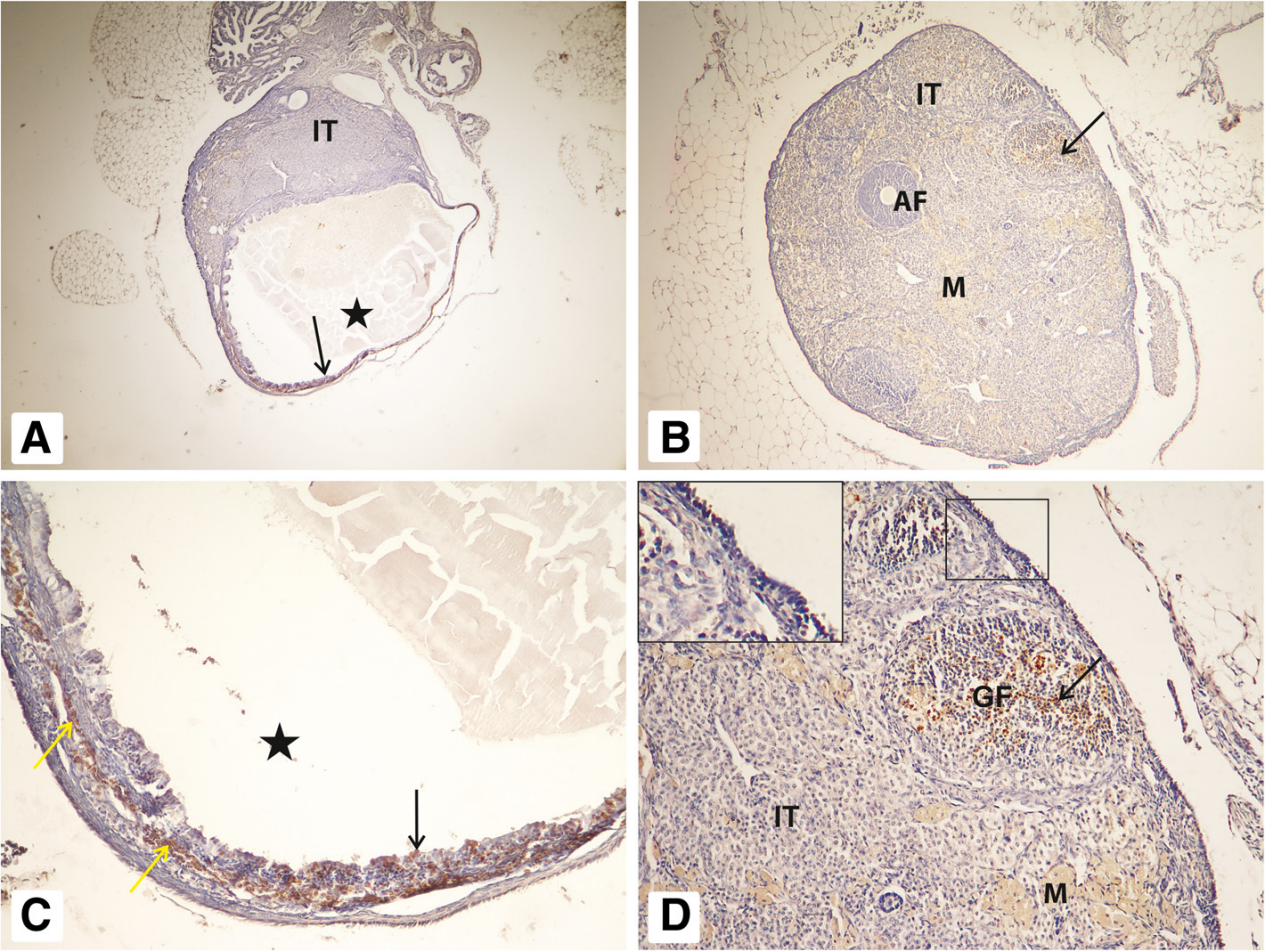
**Ovaries of WT mice.** Ovaries of 2.5-year-old WT mice fed AL **(panels A and C)** and WT mice on CR **(panels B and D)**. In WT mice on an AL diet **(panels A and C)**, a cyst (asterisk) is visible that shows a positive reaction for apoptosis in a few granulosa cells (black arrow) inside the cyst and in a few stromal cells (yellow arrow) surrounding the cyst **(panel C)**. In WT mice on caloric restriction **(panels B and D)**, single antral follicles (AF), single Graffian follicle, enlarged interstitial tissue (IT), and macrophages (M) are visible. A positive apoptotic reaction is detectable in a few granulosa cells in apical parts (cross section) of Graffian follicle **(black arrow, panels B and D)**. Magnification: panels **A** and **B** x40; panels C and D x400. Insert in **panel D** represents OSE with absence of apoptotic cells. Magnification: x800.

The ovaries of 2.5-year-old WT mice on normal AL diet were characterized by a blurred border between cortex and medulla. The majority of ovaries lacked primordial, primary, preantral, antral, and Graafian follicles (PRF, PF, PAF, AF, and GF respectively). We were able to detect single follicles in only a few ovaries. By contrast, these ovaries were characterized by abundant interstitial tissue, small degenerative follicles, and numerous hypertrophied corpora lutea. We observed that large degenerative AF developed into cysts (Figure 1, panel A) and small degenerative follicles into interstitial tissue. We also observed an increase in inflammatory cells, macrophages, and blood vessels. The cells in interstitial tissue were often surrounded by empty spaces. The surface of these ovaries was covered by a simple cuboidal epithelium. Cell apoptosis was observed in a few stromal and granulosa cells (Figure 1, panel C and Table 1).

**Table 1:** Detection of apoptosis in various ovarian cells

	**Stromal cells**	**Granulosa cells**	**Theca cells**	**Epithelial surface (OSE) cells**
**WT-AL**	+	+	–	–
**WT-CR**	+	++	–	–
**GHR-KO-AL**	–	+++	–	++
**GHR-KO-CR**	–	+++	–	++

Table legend: +++, a high number of positive cells; ++, a moderate number of positive cells, +, a low number of positive cells; **–**, an absence of apoptosis.

By contrast, the ovaries of 2.5-year-old WT mice on a CR diet appeared to be younger at the morphological level, than ovaries of WT mice on an AL diet and single follicles were present (PF, PAF, AF, and GF). The amount of interstitial tissue in these ovaries was smaller than in mice on an AL diet. The apoptotic reaction was detectable in WT mice on CR at a higher level than in AL controls in granulosa cells and in some stromal cells. In both groups, we observed atretic follicles, macrophages, and, on the surface, cuboidal epithelium (Figure 1, panels B and D and Table 1).

### Ovaries of 2.5-year-old Laron dwarf mice on AL and CR diets

**Figure 2:**
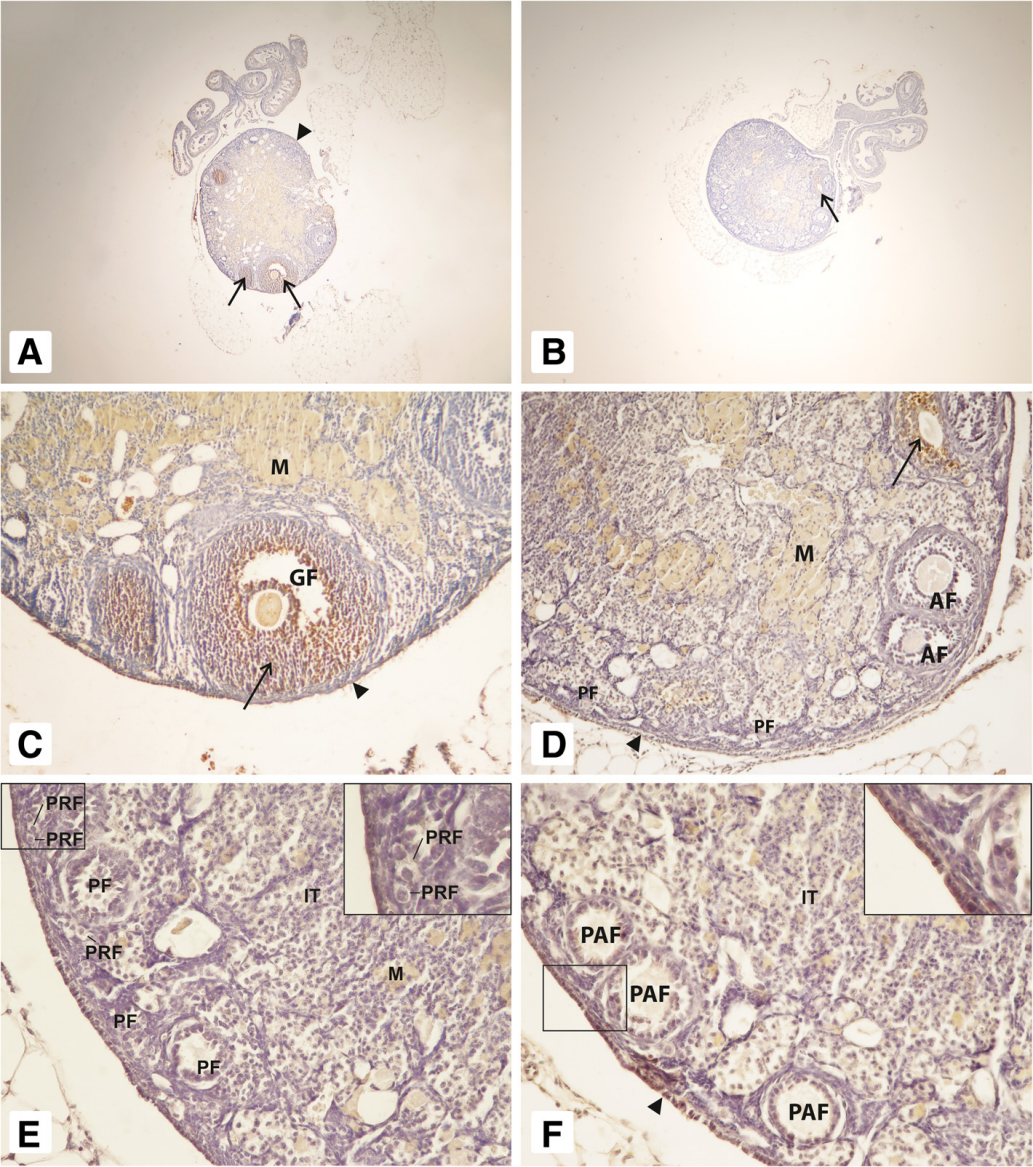
**Ovaries of GHR-KO mice.** Ovaries of 2.5-year-old GHR-KO mice fed AL **(panels A, C, and E)** and 2.5- year-old GHR-KO mice on CR **(panels B, D, and F)**. In both groups, primordial (PRF), primary (PF), preantral (PAF), antral (AF) and Graafian (GF) follicles are visible. A positive reaction for apoptosis was detectable in granulosa cells (arrow) and in ovarian surface epithelium (arrowhead) in both groups. Interstitial cells (IT), macrophages (M). Magnification: panels A and B x40; panels C and D x200; panels E and F x400. Insert in **panel E** represents primordial follicles located under OSE. Magnification: x800. Insert in **panel F** represents OSE with positive apoptotic reaction. Magnification: x800.

The morphology of ovaries in 2.5-year-old GHR-KO mice on an AL diet resembled ovaries of younger mice that are still fertile. We observed a high number of follicles at different stages of development (PRF, PF, PAF, AF, and GF) and cuboidal epithelium on the surface. However, we also detected some degenerative follicles, macrophages, and blood vessels in ovarian medulla. Moreover, the interstitial tissue (Figure 2, panels A, C, and E) was less abundant. In contrast, the ovaries of 2.5-year-old GHR-KO mice on CR had significant numbers of PAF, AF, and GF, and we observed more PRF and PF compared with GHR-KO mice on an AL diet (Figure 2, panels C and D).

The apoptosis reaction was in granulosa cells and in ovarian surface epithelium (OSE) positive at a similar level in AL and CR Laron dwarf mice. The non-specific positive reaction was observed in macrophages and in interstitial tissue (Figure 2, panels A, B, C, D, E, and F and Table 1).

## Discussion

Nutritional status is one of the major factors controlling fertility in domestic animals by affecting follicle growth and development as well as oocyte quality. As previously reported, increase of food intake in farm animals enhances insulin and IGF-1 levels which further leads to increase of number of developing follicles, decreases apoptosis and improves ovulation [2,3]. It is a beneficial effect in terms of breeding of those animals. In longer period of time increase of food intake may lead to obesity which in turn may cause fertility decreased [23]. In standard condition laboratory animals, including mice, are slightly overfed and the beneficial effect of increase of food intake and IGF-1 level would not be visible. In opposite,the effect of low level of IGF-1 was shown in Laron dwarf mice [24].This was corroborated by our previous study where we reported a lower biological age of 2-year-old Laron dwarf mouse ovaries, which have very low levels of circulating plasma IGF-1 compared with ovaries from 2-year-old WT animals [10].

In mice, the pool of ovarian follicles is established after birth as a reserve store of primordial follicles (PRF), which are available during their reproductive lifespan [4], and the positive effect of CR on reproductive lifespan has been shown both in mice and rats [4,5,11]. In these reports, the authors found increased numbers of PRF and a decrease in the numbers of atretic follicles in ovaries [4,5,11].

In our experiment, we compared morphology and the level of apoptotic cells in the ovaries of 2.5-year-old WT mice (aged mice) and 2.5-year-old GHR-KO mice. The mice were divided into four groups. We compared wild type (WT) mice on a CR diet to WT mice on a standard AL diet and GHR-KO mice on a CR diet to GHR-KO mice on a standard AL diet.

At the morphological level, we observed that the ovaries of 2.5-year-old WT mice on CR appeared to be younger than the ovaries of WT mice on an AL diet. Accordingly, the ovaries of WT mice on CR still contained some single follicles at different stages of development, which were not present in the ovaries of WT mice on an AL diet. Additionally, in many ovaries of WT mice on an AL diet, we found large cysts surrounded by interstitial tissue, and the presence of such cysts and a lack of follicles in the ovaries of aged mice was observed by other authors [25].

Furthermore, it was reported that on CR have an increased pool of primordial follicles and a decreased number of atretic follicles [4,5,11]. Li et al. [4] showed that the percentage of secondary follicles and corpora lutea decreases with duration of CR; however, the total number of surviving follicles increases with the length of CR. It is known that CR results in decreased IGF-1 levels in blood plasma and thus inhibits the process of transition from a primordial follicle to a PF and reduces follicle atresia. Thus, by lowering plasma IGF-1 levels, CR augments the reserve oocyte pool and delays the age-dependent depletion of follicles [4,26]. Gutierrez et al. [9] showed that an increase in food uptake in cattle leads to recruitment of primordial follicles and an increase in the number of developing follicles. In our study, the higher number of follicles in mice on CR indicates prolonged period of ovulatory cycles; it is beneficial for overall animal health.

We also evaluated the level of apoptosis in ovaries of WT mice on CR and on an AL diet. We found larger numbers of cells with apoptosis in follicular granulosa and stromal cells in mice on CR. By contrast, a lower number of apoptotic cells was observed in ovaries of mice on an AL diet, which might be partially explained by the lower numbers of granulosa and stromal cells.

It is known that ovarian cell death is a crucial process for maintaining ovarian homeostasis., and apoptosis in the ovary is required for efficient clearance of cells that are no longer needed, resulting in proper oocyte maturation [1,16,17,19-21]. In the processes of follicular recruitment and maturation, IGF-1 plays a key role: the high level of this factor increases recruitment and improves survival of developing follicles, while low levels of systemic IGF-1 correlates with lower levels of recruitment and increased apoptosis. Absence of GH signaling in Laron dwarf mice results in decrease in circulating IGF-1 and also decrease in ovarian IGF-1 mRNA levels. These levels may be sufficient to allow development of reduced number of functional preantral follicles and reduced litter size [24]. It is known that the IGF-1 level is decreased during CR [6], which may explain the higher apoptosis reaction in ovaries of mice on CR. In support of such a linkage, in vitro exposure of follicles to IGF-1 and GH leads to a decrease in apoptosis [27,28].

Of note, the increased level of apoptosis during CR was found not only in ovarian tissue. Muskhelishvili et al. [29] and Selman et al. [30] reported that the rate of apoptosis cell death was significantly higher in the livers of mice on CR than in AL-fed mice, which suggests that chronic CR promotes spontaneous apoptotic cell death of defective hepatocytes and may explain the significant reduction in the development of spontaneous hepatomas in these animals [29,30]. Based on this finding, an increase in apoptosis protects tissue from accumulation of damaged cells, which may be a potential source of cancer. In fact, cancer incidence decreases in rodents on CR [29,30].

The ovaries of 2.5-year-old GHR-KO mice both on an AL diet and on CR exhibited morphology typical of younger animals at reproductive age, corresponding to our recent report [10]. Interestingly, the direct comparison of ovaries of GHR-KO mice on an AL diet to GHR-KO mice on CR revealed a lower number of developed follicles in mice on CR.

In aged GHR-KO mice, we observed follicles at different stages [10]. Bachelot et al. [31] observed all follicular stages in 18-month-old GHR-KO mice and found that they were able to bear litters. It is in agreement with our recent data but our Laron mice were older (2 years old GHR-KO mice) [10]. It is known that in normal mice ovarian failure occurs at 15–17 months of age [11]. Zaczek et al. [24] noted that in adult GHR-KO mice, the number of pre-ovulatory follicles and corpora lutea was significantly reduced. Similar data were obtained by Slot et al. [32], who observed lower numbers of healthy growing primary, preantral, and antral follicles and an increased percentage of atretic follicles in GHR-KO mice compared with WT mice. The authors also noted higher numbers of primordial follicles in ovaries of GHR-KO animals [32].

In all these reports, the authors observed an increased number of atretic follicles in the ovaries of adult GHR-KO mice compared with WT mice [24,31,32]. In normal WT mice, the increase in IGF-1 concentration can modify ovarian folliculogenesis. In particular, it activates the development of preantral follicles, helps to maintain the larger pool of small antral follicles, is responsible for recruitment of more follicles to the cohort of developing follicles, and finally enables the selection process i.e. of two or more dominant follicles [33].

Since apoptosis is the cause of atresia of ovarian follicles, we should observe increased apoptosis in the ovaries of GHR-KO mice compared with normal WT animals. Danilovich et al. [22] showed that in transgenic (Tg) mice overexpressing GH, the level of apoptosis in ovarian follicles was significantly reduced compared with normal mice. On the other hand, an increase in ovarian cell apoptosis is also observed in animals on CR [29,30]. Interestingly, as reported in this paper, we observed similar numbers of apoptotic cells in GHR-KO animals on CR and on an AL diet, which can be explained by the fact that similar numbers of follicles undergo growth and maturation in ovaries of GHR-KO mice on CR and on an AL diet, and the rate of apoptosis is limited to developing follicles. Thus, this data suggests that CR do not improve the number of primordial follicles in GHR-KO mice on CR, and doesn’t influence apoptosis. Bonkowski et al. in the longevity study of Laron dwarf mice observed lack of response to CR in context of increase of lifespan in these animals. Authors suggest that longevity depends on insulin sensitivity which cannot be further increased in GHRKO mice [13,14]. We imply that in ovaries of GHR-KO mice the lack of response to CR may be due to very low level of IGF-1 which cannot be further decreased by CR.

It is known that a decrease in calorie intake improves the reproductive potential of females, but an increase in caloric intake is needed for further “usage” of these follicles in reproduction [2-5].

High levels of apoptosis in cells of ovarian surface epithelium (OSE) observed in GHR-KO mice have important implications. Specifically, apoptosis is responsible for elimination of cells, including also mutated cells in different organs, e.g., liver [29,30]. GHR-KO mice are characterized by a very low incidence of cancer [34-36], and one plausible explanation of this fact is that a low circulatory and ovarian IGF-1 level in GHR-KO keeps stem cells, including very small embryonic-like stem cells (VSELs), in quiescence and thus protects them from uncontrolled proliferation [37]. On the other hand, it is also possible that the high level of apoptosis in OSE, due to a low systemic level of IGF-1, is responsible for elimination of mutated cells and thus a lower incidence of cancer. As previously reported, OSE contains a significant number of stem cells with early embryonic developmental markers, such as SSEA-4, Oct-4, Nanog, Sox2, and c-kit [38]. In OSE, Bhartiya et al. [39] identified stem cells that express Oct-4 and SSEA-4 in addition to other pluripotent markers that correspond to the population of VSELs identified in adult tissues [40]. It is possible, that elimination of cells by apoptosis protects OSE from cancer development and corresponds to the lower incidence of cancer in GHR-KO mice, with very low levels of IGF-1 in circulating plasma.

## Conclusion

Morphological and histochemical analysis revealed a younger biological age of ovaries in 2.5-year-old WT and Laron dwarf (GHR-KO) mice on CR compared with control littermate animals at the same age on an AL diet.

## Abbreviations

AF: Antral follicles; AL: *Ad libidum*; CR: Caloric restriction; GF: Graafian follicles; GHR-KO: Growth hormone receptor knockout; IGF-1: Insulin-like growth factor 1; M: Macrophages; OSE: Ovarian surface epithelium; PAF: Preantral follicles; PF: Primary follicles; PRF: Primordial follicles; Tg: Transgenic; WT: Wild type; VSELs: Very small embryonic-like stem cells.

## Competing interests

The authors declare that they have no competing interests.

## Authors’ contributions

SSG: performed morphological and histochemical analysis of ovarian sections, found the result, and wrote the manuscript. ML: helped in planning, supervised the work, participated in morphological and histochemical analysis of ovarian sections, and helped in writing the manuscript. KP: participated in results analysis and helped in writing the manuscript. WG: helped in morphological and histochemical analysis of ovarian sections, found the result, and corrected the manuscript. BR: corrected the manuscript. MM: Performed the experiment with caloric restriction. OA: performed the experiment with caloric restriction. JJK: provided Laron dwarf mice and approved the manuscript. AB: performed the experiment with caloric restriction and approved a final version of the manuscript. MK: obtained the ovarian tissue. MR: helped in planning, supervised the work, helped in writing the manuscript, and corrected the final version of manuscript. All authors have read and approved the final manuscript.

## References

[B1] Hussein MR Apoptosis in ovary: molecular mechanisms Human Reprod Update 2005 11 162 178 10.1093/humupd/dmi001 15705959

[B2] Amstrong DG Gong JG Gardner JO Baxter G Hogg CO Webb R Steroidogenesis in bovine granulose cells: the effect of short-term changes in dietary intake Reproduction 2002 123 371 378 10.1530/rep.0.1230371 11882014

[B3] Amstrong DG Gong JG Webb R Interactions between nutrition and ovarian activity in cattle: physiological, cellular and molecular mechanisms Reprod Suppl 2003 61 403 414 14635951

[B4] Li L Fu YC Xu JJ Chen XC Lin XH Luo LL Caloric restriction promotes the reproductive capacity of female rats via modulating the level of insulin-like growth factor-1 (IGF-1) Gen Comp Endocrinol 2011 174 232 237 10.1016/j.ygcen.2011.09.005 21945120

[B5] Nelson J Gosden RG Felicio LS Effect of dietary restriction on estrous cyclicyty and follicular reserves in aging C57BL/6J mice Biol Reprod 1985 32 515 522 10.1095/biolreprod32.3.515 4039610

[B6] Masoro EJ Overview of caloric restriction and ageing Mech Ageing Dev 2005 126 913 922 10.1016/j.mad.2005.03.012 15885745

[B7] Osborne TB Mendel LB Ferry EL The effect of retardation of growth upon the breeding period and duration of life in rats Science 1917 45 294 295 10.1126/science.45.1160.294 17760202

[B8] Masoro EJ McCarter RJM Katz MS McMahan CA Dietary restriction alters the characteristics of glucose fuel use J Gerontol Biol Sci 1992 47 B202 B208 10.1093/geronj/47.6.B202 1430849

[B9] Gutierrez CG Oldham J Bramley TA Gong JG Campbell BK Webb R The recruitment of ovarian follicles is enhanced by increased dietary intake in heifers J Anim Sci 1997 75 1876 1884 9222845 10.2527/1997.7571876x

[B10] Słuczanowska-Głąbowska S Laszczyńska M Piotrowska K Głąbowski W Kopchick JJ Bartke A Kucia M Ratajczak MZ Morphology of ovaries in laron dwarf mice, with low circulating plasma levels of insulin-like growth factor-1, and in bovine GH-transgenic mice, with high circulating plasma levels of IGF-1 J Ovarian Res 2012 5 18 10.1186/1757-2215-5-18 22747742 PMC3583234

[B11] Selesniemi K Lee HJ Tilly JL Moderate caloric restriction initiated in rodents during adulthood sustains function of the female reproductive axis into advanced chronological age Aging Cell 2008 7 622 629 10.1111/j.1474-9726.2008.00409.x 18549458 PMC2990913

[B12] Laron Z Laron syndrome (primary growth hormone resistance or insensitivity): the personal experience 1958–2003 J Clin Endocrinol 2004 89 1031 1044 10.1210/jc.2003-031033 15001582

[B13] Bonkowski MS Rocha JS Masternak MM Regaiey KA Bartke A Targeted disruption of growth hormone receptor interferes with the beneficial actions of calorie restriction PNAS 2006 103 7901 7905 10.1073/pnas.0600161103 16682650 PMC1458512

[B14] Bonkowski MS Dominici FP Arum O Rocha JS Al Regaiey KA Westbrook R Spong A Panici J Masternak MM Kopchick JJ Bartke A Disruption of growth hormone receptor prevents calorie restriction from improving insulin action and longevity PLoS One 2009 4 e4567 10.1371/journal.pone.0004567 19234595 PMC2639640

[B15] Zhou Y Xu BC Maheshwari HG He L Reed M Lozykowski M Okada S Cataldo L Coschigamo K Wagner TE Baumann G Kopchick JJ A mammalian model for Laronsyndrome produced by target disruption of the mouse growth hormone receptor/binding protein gene (Laron mouse) Proc Natl Acad Sci 1997 94 13215 13220 10.1073/pnas.94.24.13215 9371826 PMC24289

[B16] Brodowska A Laszczyńska M Starczewski A Apoptosis In ovaria cells In postmenopausalwoman Folia Histochem Cytobiol 2007 45 99 105 17597023

[B17] Hułas-Stasiak M Gawron A Follicular atresia in the prepubertal spiny Mouse (Acomyscahirinus) ovary Apoptosis 2011 16 967 975 10.1007/s10495-011-0626-9 21739276

[B18] Hsueh AJ Eisenhauer K Chun SY Hsu SY Billig H Gonadal cell apoptosis Recent ProgHorm Res 1996 51 433 455 8701090

[B19] Amsterdam A Sasson R Keren-Tal I Aharoni D Dantes A Rimon E Land A Cohen T Dor Y Hirsh L Alternative pathways of ovarian apoptosis: death for life Biochem Pharmacol 2003 66 1355 1362 10.1016/S0006-2952(03)00485-4 14555209

[B20] Amsterdam A Keren-Tal I Aharoni D Dantes A Land-Bracha A Rimon E Sasson R Hirsh L Steroidogenesis and apoptosis in the mammalian ovary Steroids 2003 68 861 867 10.1016/j.steroids.2003.09.003 14667978

[B21] Brodowska A Laszczyńska M Starczewski A Rola apoptozy w komórkach jajnika Post Biol Kom 2006 33 35 44

[B22] Danilovich NA Bartke A Winters TA Ovarian follicle apoptosis in bovine growth hormone transgenic mice Biol Reprod 2000 62 103 107 10.1095/biolreprod62.1.103 10611073

[B23] Bermejo-Alvarez P Rosenfeld CS Roberts RM Effect of maternal obesity on estrous cyclicity, embryo development and blastocyst gene expression in a mouse model Hum Reprod 2012 27 3513 3522 10.1093/humrep/des327 23001779 PMC3501243

[B24] Zaczek D Hammond J Suen L Wandji S Service D Bartke A Chandrashekar V Coschigano K Kopchick J Impact of growth hormone resistance on female reproductive function: new insights from growth hormone receptor knockout mice Biol Reprod 2002 67 1115 1124 10.1095/biolreprod67.4.1115 12297526

[B25] Davis BJ Dixon D Herbert RA Maronpot RR Ovary, oviduct, uterus, cervix and vagina Pathology of the mouse 1999 St Louis, USA: Cache River Press 409 443

[B26] Yu Y Li W Han Z Luo M Chang Z Tan J The effect of follicle-stimulating hormone on follicular development, granulosa cell apoptosis and steroidigenesis and its mediation by insulin-like growth factor-I in the goat ovary Theriogenology 2003 60 1691 1704 10.1016/j.theriogenology.2003.08.001 14580651

[B27] Chun SY Eisenhauer KM Minami S Billig H Perlas E Hsueh AJ Hormonal regulation of apoptosis in early antral follicles: Follicle-stimulating hormone as a major survival factor Endocrinology 1996 137 1447 1456 10.1210/en.137.4.1447 8625923

[B28] Sharma GT Dubey PK Kumar GS Effects of IGF-1, TGF-α plus TGF-β_1_ and bFGF on *in vitro* survival, growth and apoptosis in FSH-stimulated buffalo (Bubalisbubalus) preantral follicles Growth Hormone and IGF Research 2010 20 319 325 10.1016/j.ghir.2010.05.001 20726112

[B29] Muskhelishvili L Hart RW Turturro A James J Age-related changes in the intrinsic rate of apoptosis in livers of diet-restriction and *ad libitum*-fed B6C3F1 mice Am J Pathol 1995 147 20 24 7604880 PMC1869890

[B30] Selman C Kendaiah S Gredilla R Leeuwenburgh C Increased hepatic apoptosis during short-term caloric restriction is not associated with an enhancement in caspase levels Exp Gerontol 2003 38 897 903 10.1016/S0531-5565(03)00091-3 12915211

[B31] Bachelot A Monger P Imbert-Bolloré P Coshigano K Kopchick JJ Kelly PA Binart N Growth Hormone is required for ovarian follicular growth Endocrinology 2002 143 4104 4112 10.1210/en.2002-220087 12239122

[B32] Slot KA Kastelijn J Bachelot A Kelly PA Binart N Teerds KJ Reduced recruitment and survival of primordial and growing follicles in GH receptor-deficient mice Reproduction 2006 131 525 532 10.1530/rep.1.00946 16514195

[B33] Silva JRV Figuiredo JR Van den Hurk R Involvment of growth hormone (GH) and insulin-like growth factor (IGF) system in ovarian folliculogenesis Theriogenology 2009 71 1193 1208 10.1016/j.theriogenology.2008.12.015 19193432

[B34] Laron Z The GH-IGF1 axis and longevity. The paradigm of IGF1 deficiency Hormones 2008 7 24 27 18359741 10.14310/horm.2002.1111034

[B35] Bartke A Sun LY Longo V Somatotropic signaling: trade-offs between growth, reproductive development and longevity Physiol Rev 2013 93 571 598 10.1152/physrev.00006.2012 23589828 PMC3768106

[B36] Ikeno Y Hubbard GB Lee S Cortez LA Lew CM Webb CR Berryman DE List EO Kopchick JJ Bartke A Reduced incidence and delayed occurrence of fatal neoplastic diseases in growth hormone receptor/binding protein knockout mice J Gerontol A BiolSci Med Sci 2009 64 522 529 10.1093/gerona/glp017 PMC2667132 19228785

[B37] Ratajczak J Shin DM Wan W Liu R Masternak MM Piotrowska K Wiszniewska B Kucia M Bartke A Ratajczak MZ Higher number of stem cells in the bine marrow of circulating low Igf-1 level Laron dwarf mice – novel viev on Igf-1, stem cells and aging Leukemia 2011 25 729 733 10.1038/leu.2010.314 21233833 PMC3746340

[B38] Virant-Klun I Zech N Rozman P Vogler A Cyjeticanin B Klemene P Malicev E Meden-Vrtovec H Putative stem cells with embryonic character isolated from the ovarian surface epithelium of women with no naturally present follicles and oocytes Differentiation 2008 76 843 856 10.1111/j.1432-0436.2008.00268.x 18452550

[B39] Bhartiya D Unni S Parte S Anand S Very small embryonic-like stem cells: implications in reproductive biology Bio Med Res Int 2013 682326 doi: 10.1155/2013/682326 10.1155/2013/682326 PMC3586435 23509758

[B40] Zuba-Surma EK Kucia M Wu W Klich I Lillard J Ratajczak J Ratajczak MZ Very small embryonic-like stem cells are present in adult murine organs: imagestream-based morphological analysis and distribution studies Cytometry A 2008 73A 1116 1127 10.1002/cyto.a.20667 18951465 PMC2646009

